# Long-term results of transpedicle body augmenter in treating burst fractures

**DOI:** 10.4103/0019-5413.41865

**Published:** 2008

**Authors:** Dilbans Singh Pandher, Rajesh Kapila, Ajay Gupta

**Affiliations:** *Department of Orthopedics, Oxford Super-specialty Hospital, Jallandhar, India*; 1 *Department of Orthopedics, Govt. Medical College, Amritsar, India*

Sir,

We have read with interest the article “Long-term results of transpedicle body augmenter in treating burst fractures” by Li *et al*.^1^ This is an interesting article, which has tried to address the issue of short-segment fixation very well by comparing the transpedicle augmenter with a control group operated by same surgeon. Authors have been honest by elaborately addressing their shortcoming in the discussion.

Postoperative protocol has a significant importance in any kind of spinal surgery. However, the study does not mention the details of the postoperative protocol. Since delayed ambulation after short-segment fixation is known to give good results,^2^ authors need to mention the postoperative protocol with specific mention of mobilization and ambulation. Moon *et al.*,^2^ have demonstrated that short-segment fixation without posterolateral fusion is an effective procedure for compression and burst fractures if the postoperative mobilization is delayed by two to four weeks. It would be better if the results of transpedicle body augmenter could be compared with long-segment fixation.

The study reports mean time of surgery as 63.3 ± 13.2 min and 63.1 ± 17.2 min for the augmenter and control groups respectively. Does this mean that no additional time is required for the following: (1) preparation of bilateral pedicle tunnels to the fractured vertebra with awl; followed by serial custom-made trials to prepare for TpBA passage; (2) harvesting bone graft from iliac crest; (3) filling the vertebral body with autologous bone graft; (4) inserting the augmenter through pedicle; and (5) filling the pedicle tunnel space with bone graft.

Similarly, the blood loss reported for the augmenter group and control group is 227 ± 72 cc and 242 ± 89 cc respectively, which means the blood loss is less in the group where two additional pedicle tunnels were made and bone graft was harvested from the iliac crest. If it is so, authors need to justify why.

In the ‘Materials and Methods’ section, it is reported that flexion and extension X-rays were taken after one year and at final visit. The purpose of flexion-extension X-rays is not mentioned in the article. Generally, flexion-extension X-rays are required for judging the bony union after the spinal fusion is attempted. However, in this study the vertebral body augmentation is compared with short segment fixation alone. So authors should comment upon what additional information was acquired from flexion-extension X-rays once the anterior body height and kyphosis angle was measured on neutral thoracolumbar radiographs.
